# 1519. Increased incidence of colorectal cancer in Veteran Health Administration hospital patients with pyogenic liver abscess: A matched cohort study

**DOI:** 10.1093/ofid/ofac492.081

**Published:** 2022-12-15

**Authors:** Ian Kidder, Michihiko Goto, Hiroyuki Suzuki

**Affiliations:** University of Iowa Hospitals and Clinics, Iowa City, IA; University of Iowa/Iowa City VAMC, Iowa City, Iowa; University of Iowa Carver College of Medicine, Iowa City, Iowa

## Abstract

**Background:**

Colorectal cancer (CRC) remains the third leading cause of death in the United States (US), and incidence has increased among patients < 50 years of age over the last few decades. Although many patients with early CRC remain asymptomatic, infections such as *Streptococcus gallolyticus* bacteremia, can be an early finding. There is increasing evidence that pyogenic liver abscesses (PLA) may also be associated with CRC. However, studies of US populations remain limited.

**Methods:**

We retrospectively evaluated patients with a diagnosis of PLA at Veteran Health Administration hospitals across the US from 2003 to 2020. For each case, up to three uninfected controls matched for age (+/- 5), gender, and healthcare facilities at the time of index infection from the outpatient population were selected. PLA patients and matched controls were followed for ten years. Our primary outcome was a new diagnosis of CRC after index infection. Stratified cumulative incidence function plots with Gray’s test for equality were used to examine CRC incidence. Then we used Fine-Gray sub-distribution hazard regression models with interaction terms using the logarithm of time to estimate cause-specific time-dependent hazard ratios (HRs) for CRC incidence while accounting for mortality.

**Results:**

We compared 8,294 PLA patients to 23,111 matched controls. Median age was 65.7 in PLA patients and 66.0 in matched controls. The median number of colonoscopies was ten in PLA patients and five in matched controls over the study period (p < 0.001). Our analysis revealed a 1.9% incidence of new CRC in the liver abscess group, compared to 0.8% in the matched cohort (p < 0.001, Figure 1). The multivariate sub-distribution hazard regression model showed higher time-dependent HRs of CRC up to two years from diagnosis of PLA but not higher afterward (Table 1).

Incidence of colorectal cancer (CRC) in patients with liver abscess (dashed yellow line) compared to patients with no liver abscess (solid blue line). Our analysis revealed a 1.9% incidence of CRC in patients with liver abscess compared to 0.8% in the matched control cohort.

**Conclusion:**

We found a significantly higher incidence of CRC for patients with PLA, especially during the first two years from diagnosis, although some of our findings may be explained by increased screening around the time of diagnosis. We believe our findings support the importance of CRC screening after the diagnosis of PLA.

**Disclosures:**

**Michihiko Goto, MD MSCI**, Merck & Co.: Grant/Research Support.

